# Clinical Outcome of Patients with Acute Periprosthetic Joint Infections Caused by *Pseudomonas aeruginosa* Compared to Other Gram-Negative Bacilli

**DOI:** 10.3390/microorganisms13040904

**Published:** 2025-04-14

**Authors:** Wai-Yan Liu, Johannes G. E. Hendriks, Robin W. T. M. van Kempen, Walter van der Weegen, Wim H. C. Rijnen, Jon H. M. Goosen, Babette C. van der Zwaard, Yvette Pronk, Wierd P. Zijlstra, Bas L. E. F. ten Have, Joris J. W. Ploegmakers, Marjan Wouthuyzen-Bakker

**Affiliations:** 1 Department of Orthopaedic Surgery & Trauma, Catharina Hospital, 5623 EJ Eindhoven, The Netherlands; 2 Department of Orthopaedic Surgery & Trauma, Máxima MC, 5631 BM Eindhoven, The Netherlands; 3 Sports & Orthopedics Research Center, Anna Hospital, 5664 EH Geldrop, The Netherlands; 4 Department of Orthopaedic Surgery, Radboud University Medical Center, 6525 GA Nijmegen, The Netherlands; 5 Department of Orthopaedic Surgery, Sint Maartenskliniek, 6532 SZ Nijmegen, The Netherlands; 6 Department of Orthopedics, Jeroen Bosch Hospital, 5223 GZ ’s-Hertogenbosch, The Netherlands; 7 Research Department of Orthopedic Surgery, Kliniek ViaSana, 5451 AA Mill, The Netherlands; 8 Department of Orthopedic Surgery, Medical Center Leeuwarden, 8934 AD Leeuwarden, The Netherlands; 9 Department of Orthopedics, Martini Hospital, 9728 NT Groningen, The Netherlands; 10 Department of Orthopaedic Surgery, University Medical Center Groningen, University of Groningen, 9713 GZ Groningen, The Netherlands; 11 Department of Medical Microbiology and Infection Prevention, University Medical Center Groningen, University of Groningen, 9713 GZ Groningen, The Netherlands; m.wouthuyzen-bakker@umcg.nl

**Keywords:** debridement, treatment, antibiotics, outcome

## Abstract

*Pseudomonas aeruginosa* is considered as more difficult to treat than other Gram-negatives in patients with acute periprosthetic joint infections (PJIs). However, clinical data to support this hypothesis are lacking. This retrospective multicenter cohort study included 39 patients with acute PJIs caused by *P. aeruginosa* and 84 control patients with another Gram-negative bacillus (i.e., Enterobacterales). Both groups were managed by surgical debridement, antibiotics, and implant retention (DAIR). Treatment failure within one-year follow-up was defined as prosthesis extraction, a clinical need for suppressive antibiotic treatment and/or PJI-related death. Distribution of affected joints, and revision versus primary arthroplasties, did not differ between groups. Most PJIs were polymicrobial (87% in cases, 81% in control patients, *p* = 0.451). Surgical and antibiotic management was similar between groups. Treatment failure did not differ between groups: 5/39 cases (12.8%) and 14/84 control patients (16.7%, *p* = 0.610). An acceptable success rate of acute PJI caused by *P. aeruginosa* when treated with DAIR was observed. This success rate did not differ compared to PJIs caused by Enterobacterales. Therefore, *P. aeruginosa* should not be considered a more difficult to treat microorganism compared to other Gram-negatives. No additional surgical or antimicrobial interventions are needed when patients can be treated with a fluoroquinolone.

## 1. Introduction

The development of a periprosthetic joint infection (PJI) is a major complication that can occur after primary or revision arthroplasty [1]. In acute PJIs, the main surgical strategy is thorough surgical debridement followed by a long duration of antibiotic treatment, with the aim to retain the original implant (DAIR) [2]. In case this treatment strategy fails, the implant often needs to be extracted and replaced by a new prosthesis. The success rate of a DAIR procedure varies between 55% and 90% [3] and depends on several factors, like the time between the index surgery and the DAIR procedure [4], the type of infection [5], host factors [6], and the causative microorganism [7].

PJIs are caused by Gram-positive microorganisms in the majority of cases, but Gram-negative bacilli are present in up to 10 to 15% of cases, often as part of a polymicrobial infection [8]. The incidence of Gram-negative PJIs is rising according to some reports [9] and therefore, having knowledge on the clinical outcome of different types of Gram-negative bacilli is important to ultimately improve future strategies. In general, *Pseudomonas aeruginosa* as Gram-negative bacillus is considered a difficult to treat microorganism in PJI due to its characteristic of being a strong biofilm producer [10,11]. Indeed, failure rates of up to 75% have been described in PJIs caused by *Pseudomonas aeruginosa* when treated with DAIR [12]. However, clinical data comparing the outcome of *Pseudomonas aeruginosa* PJIs compared to other Gram-negative bacilli are lacking.

Therefore, the aim of our study is to evaluate a cohort of patients with an early acute PJI and compare the clinical outcome of those cases caused by *Pseudomonas aeruginosa* and those caused by Enterobacterales.

## 2. Materials and Methods

### 2.1. Study Design

In this retrospective multicenter cohort study, data from 2 regional cohorts (Regional Infection Cohort (RIC) and Northern Infection Network Joint Arthroplasty (NINJA)) were combined. A total of 9 hospitals participated in this study. The study protocol was reviewed and approved by each institutional review board.

### 2.2. Study Population

Both regional cohorts comprised data from patients with an early acute (postoperative) knee or hip PJI treated with DAIR (RIC between 2014 and 2018 [13] and NINJA between 2008 and 2015 [4]). Early acute PJIs were defined as those occurring within 3 months after index arthroplasty. PJI was defined solely based on microbiological criteria. Patients were selected for inclusion when intraoperative cultures isolated *P. aeruginosa* (cases) or Enterobacterales as Gram-negative bacillus (i.e., *Escherichia coli*, *Escherichia vulneris*, *Klebsiella oxytoca*, *Klebsiella pneumoniae*, *Proteus mirabilis*, or *Proteus vulgaris*) (control patients). One positive intraoperative tissue culture with *Pseudomonas aeruginosa* or Enterobacterales was considered sufficient for inclusion [14].

### 2.3. Data Collection

Databases from both cohorts comprised patient characteristics (e.g., age, sex, body mass index), surgical parameters (e.g., primary or revision arthroplasty, hip or knee arthroplasty), inflammatory parameters, microbiological data of cultures, treatment of PJI, and outcome at one year. A clinical visit at one-year follow-up was conducted to evaluate the PJI treatment.

### 2.4. Surgical and Antimicrobial Treatment

During the DAIR procedure, multiple tissue samples were obtained for cultures (at least 5) from synovium, capsule, and interface. This was followed by removal of modular prosthetic components to allow for proper joint debridement, provided that it does not pose a risk of damaging the prosthesis. Thorough debridement and irrigation were performed using pulse lavage with at least 6 L NaCl before exchangeable components were replaced [13,15]. When mobile components were not available during DAIR, they were extracted if possible to allow sufficient debridement of the joint cavity, aggressively cleansed and re-inserted. A second DAIR was only performed in case of persistent clinical signs of infection or recurrent wound leakage without any alternative explanation, provided that the soft tissues are intact.

Antibiotic treatment was determined in consultation with a medical microbiologist and/or internist infectiologist. In general, patients were treated with antibiotics for 3 months in total.

### 2.5. Outcomes

The primary outcome was treatment failure within one year of follow-up, which was defined as prosthesis extraction, the clinical need for suppressive antibiotic treatment and/or PJI-related death. A second DAIR was not considered a treatment failure. Treatment success was defined as infection control with retention of the implant and without the need for suppressive antibiotics.

### 2.6. Statistical Analysis

Continuous variables were summarized as medians and ranges and categorical variables as percentages of the total sample for that variable. Mann–Whitney U test, Chi-square test, or Fisher’s exact test were used to compare the differences between groups. Subgroup analyses were performed to assess differences in treatment failure between monomicrobial and polymicrobial PA cases and between monomicrobial and polymicrobial control patients. A *p*-value of <0.05 was considered as statistically significant. Statistical analysis was performed using IBM SPSS Statistics^®^ version 28 (Armonk, NY, USA).

## 3. Results

### 3.1. Characteristics Cases and Control Patients

A total of 39 cases and 84 control patients were included in the study. From the control patients, *Escherichia coli* was isolated in 46%, *Proteus* species in 36% and *Klebsiella* species in 18%. Table 1 shows the baseline characteristics of both groups. Median age was 75 years (64–82) in cases and 74 years (65–83) in control patients (*p* = 0.598). No differences in baseline characteristics were found, except for the use of immunosuppressants, which was higher in the cases with *Pseudomonas aeruginosa*.

Table 2 shows the clinical presentation and surgical and antibiotic treatment for both groups. Overall, the clinical presentation, rate of polymicrobial infections, and the timing from index surgery to DAIR was comparable between groups. Lower inflammatory serum parameters before the DAIR procedure were observed in cases with *P. aeruginosa* compared to control patients. Overall, surgical and antimicrobial management was similar between both groups. Median days between index surgery and first DAIR did not differ between groups (*p* = 0.465). Control patients received ciprofloxacin to a lesser extent compared to cases (61% versus 90%, *p* = 0.019) and combination therapy was more often used in control patients (31% versus 13%, *p* = 0.014).

In Table 3, cultured microorganisms during the first DAIR are presented. Other microorganisms were isolated in 22 polymicrobial cases (56%) and 50 control patients (60%). In control patients, *Escherichia coli* and *Proteus mirabilis* were isolated in 37 subjects (45%) and 36 subjects (43%), respectively. The combination of cultured microorganisms in 34 polymicrobial cases and 68 control patients are presented in Appendix A. In all patients fluroquinolones was used in the oral antibiotic treatment after the first DAIR procedure Table 4). Rifampicin was the second most used oral antibiotic in both groups.

### 3.2. Clinical Outcome

Treatment failure at one year did not differ between the groups: 5 out of 39 (12.8%) *P. aeruginosa* cases failed as opposed to 14 out of 84 (16.7%) control patients with a PJI due to Enterobacterales (*p* = 0.610; odds ratio of 0.7, 95% confidence interval 0.244–2.21). Prosthesis extraction during follow up was performed in three cases (7.7%) and in nine control patients (10.7%, *p* = 1.00). Suppressive antibiotics was administered to two control patients (*p* = 1.00). Two PJI-related deaths were observed in cases and three in control patients (*p* = 0.652). Overall treatment success was 85% for all subjects. Monomicrobial PA cases (*n* = 5) did not fail at one-year follow-up. Monomicrobial PA cases did not significantly differ in clinical outcome at one year after treatment compared to polymicrobial PA cases (*p* = 1.00). In addition, monomicrobial control patients did not differ in clinical outcome compared to polymicrobial control patients (*p* = 0.733).

## 4. Discussion

Our study demonstrates that an early acute PJI caused by *Pseudomonas aeruginosa* is not more difficult to treat compared to a PJI caused by Enterobacterales when treated by DAIR. Our results are strengthened by the fact that both groups received the same surgical and antimicrobial strategy and, nevertheless, demonstrated similar outcomes.

Our results are in concordance with some previous reports on *Pseudomonas* PJIs. Acceptable clinical outcomes have been described, with success rates varying between 63 and 80% when treated by DAIR [16,17]. Gonzalez et al. [18] performed a systematic review of the literature and evaluated 593 PJIs caused by Gram-negatives. The authors demonstrated that acute PJIs caused by *Pseudomonas* species were associated with a lower instead of a higher rate of failure compared to the *Proteus* species and *E. coli*. Although we also found higher inflammatory parameters in patients with an Enterobacterales compared to *Pseudomonas*, these clinical features were not associated with a worse outcome in our analysis. One study reports a very high failure rate for *Pseudomonas* PJIs. Shah et al. [12] evaluated 102 episodes of *Pseudomonas* PJIs of whom 18 were treated with DAIR. Those patients treated with DAIR had a success rate of only 26% after a follow-up period of two years. The high failure rate described in this study might be due to the longer follow-up period, a relatively long duration of symptoms prior to DAIR (more than two weeks), the quality of surgical debridement, the definition of failure, and the relatively high rate of quinolone resistant *Pseudomonas* strains (20%). Fluoroquinolones are known for their excellent antibiofilm activity [19,20,21] and observational studies demonstrate that Gram-negative PJIs treated with fluoroquinolones are associated with higher success rates [22,23]. In our cohort of *Pseudomonas* PJIs, 90% were treated with ciprofloxacin as an oral antibiotic after an induction period with a beta-lactam. This percentage was lower in our control group with Enterobacterales as only 62% of them were treated with ciprofloxacin.

Despite *Pseudomonas’* greater biofilm-forming ability, the combination of effective debridement, targeted antibiotic therapy, and host factors likely contributes to similar treatment outcomes when compared to Enterobacterales PJIs. In our study, *Pseudomonas* remained susceptible to a range of potent antimicrobial therapies, including beta-lactams, aminoglycosides, and fluoroquinolones. In addition, the success of debridement depends on factors such as infection chronicity, host immune response, and bacterial load rather than biofilm formation alone. Surgical debridement physically removes infected and necrotic tissue, reducing the bacterial load and disrupting biofilms. Even though *Pseudomonas* forms robust biofilms, their removal via debridement may be sufficient to allow antibiotics to eliminate residual bacteria effectively.

Our study should be received in light of a few limitations. First, the large majority of patients had a polymicrobial infection, which makes it difficult to solely conclude about the outcome of one specific microorganism. However, the rate of polymicrobial infections was similar between both groups, and the clinical outcome was the same. Moreover, subgroup analysis demonstrated no differences in clinical outcome between monomicrobial and polymicrobial infections within each group. Second, we cannot fully exclude the possibility that *Pseudomonas* PJIs received a more aggressive treatment, since *Pseudomonas* infections are often perceived by clinicians as difficult to treat. However, all the parameters investigated, e.g., the rate of second DAIRs, exchange of mobile components, use of local antibiotics, use of systemic antibiotic duotherapy, and antibiotic treatment duration, were not higher in cases with *Pseudomonas*. The sample size of included patients, especially for *Pseudomonas* cases, was relatively limited. This may reduce the generalizability of our findings. In addition, correction for potential confounders was not possible due to the limited sample size and the number of events per variable [24]. Future studies with larger sample sizes may identify potential predictors of treatment failure. Although the majority of failures (>95%) fail within the first year after DAIR [25], a one-year follow-up may not capture late failures or recurrence of PJIs. Therefore, late failures could potentially be underestimated. Studies evaluating a longer follow-up period (at least two years) are recommended to improve the external validity of the results.

## 5. Conclusions

In conclusion, based on our analysis, *Pseudomonas aeruginosa* PJIs do not seem more difficult to treat compared to other Gram-negative PJIs when treated with ciprofloxacin. No additional surgical or antimicrobial interventions are needed when patients can be treated with a fluoroquinolone.

## Figures and Tables

**Table 1:** Baseline characteristics.

Characteristic	Cases	Control Patients	*p*-Value
Median age (IQR), years	75 (64–82)	74 (65–83)	0.598
Median BMI (IQR), kg/m^2^	29 (25–35) ^#^	29 (26–36) ^^^	0.920
Male sex, *n* (%)	18 (46)	37 (44)	0.848
ASA classification			
ASA I, *n* (%)	2 (5.1)	8 (9.5)	0.665
ASA II, *n* (%)	21 (53.8)	47 (56.0)	
ASA III–IV, *n* (%)	16 (41.1)	29 (34.5)	
Involved joint			
Knee arthroplasty, *n* (%)	13 (33.3)	24 (28.6)	0.674
Hip arthroplasty, *n* (%)	26 (66.7)	60 (71.4)	
Type of arthroplasty			
Primary arthroplasty, *n* (%)	26 (66.7)	68 (81.0)	0.110
Revision arthroplasty, *n* (%)	13 (33.3)	15 (17.9)	
Comorbidities			
Diabetes mellitus, *n* (%)	4 (10.3)	14 (16.7) ^‡^	0.420
Ischemic heart disease, *n* (%)	2 (5.1)	14 (16.7) ^†^	0.089
Immunosuppressant, *n* (%)	5 (12.8)	1 (1.2) ^$^	0.013
Hypertension, *n* (%)	18 (46.2)	40 (47.6) ^¶^	0.849
Rheumatoid arthritis, *n* (%)	3 (7.7)	8 (9.5) ^¥^	1
Heart failure, *n* (%)	5 (12.8)	13 (15.5) ^§^	0.789
Use of acenocoumarol, *n* (%)	11 (28.2)	18 (21.4) ^¥^	0.496
Chronic obstructive pulmonary disease, *n* (%)	5 (12.8)	15 (17.9) ^†^	0.603
Malignancy, *n* (%)	7 (17.9)	14 (16.7) ^£^	1
Smoking, *n* (%)	7 (17.9)	9 (10.7) ^∨^	0.388
Alcohol use, *n* (%)	13 (33.3)	26 (31.0) ^∨^	0.838

Notes: ^#^ 1 missing values; ^¶^ 4 missing values; ^^^ 5 missing values; ^‡^ 6 missing values; ^†^ 7 missing values; ^§^ 9 missing values; ^∨^ 11 missing values; ^¥^ 13 missing values; ^£^ 14 missing values; ^$^ 17 missing values.

**Table 2:** Clinical presentation and surgical and antibiotic treatment.

Characteristic	Cases	Control Patients	*p*-Value
Clinical Presentation			
Purulence, *n* (%)	5 (12.8)	13 (15.5)	0.790
Persistent wound leakage, *n* (%)	33 (84.6)	69 (82.1)	0.803
Redness, *n* (%)	19 (48.7)	40 (47.6)	1
Fever (temperature > 38 degrees Celsius), *n* (%)	3 (7.7)	19 (22.6)	0.074
Wound dehiscence, *n* (%)	3 (7.7)	6 (7.1)	1
Hematoma, *n* (%)	2 (5.1)	7 (8.3)	0.718
Septic shock, *n* (%)	1 (2.6)	7 (8.3)	0.279
Necrosis, *n* (%)	3 (7.7)	2 (2.4)	0.325
CRP before first DAIR, mg/L median (IQR)	48 (8–97)	75 (27–172) ^$^	0.014
Leucocytes before first DAIR × 10^9^/L median (IQR).	8.5 (8–11) ^†^	10 (9–14) ^‡^	0.046
Days between index surgery and first DAIR, *n* (IQR)	16 (13–22)	17 (14–22)	0.465
Polymicrobial, *n* (%)	34 (87.2)	68 (81)	0.451
Surgical and antibiotic treatment			
Second DAIR *n* (%)	12 (30.8)	39 (46.4)	0.118
Local gentamicin usage during first DAIR, *n* (%)	15 (38.5)	37 (44.0) ^$^	0.561
Modular component exchange during first DAIR, *n* (%)	16 (41)	30 (35.7) ^†^	0.693
Intravenous antibiotic treatment <2 weeks, *n* (%)	21 (54)	39 (46)	0.561
Administration of ciprofloxacin, *n* (%)	35 (89.7)	51 (60.7) ^#^	0.019
Use of combination therapy longer than 3 days, *n* (%)	5 (12.8)	26 (31) ^^^	0.014
Days of antimicrobial therapy, median (IQR)	91 (18.3–95.5) ^†^	92 (20–124.5) ^^^	0.221

Notes: ^$^ 1 missing value; ^†^ 3 missing values; ^‡^ 19 missing values; ^#^ 11 missing values; ^^^ 12 missing values.

**Table 3:** Cultured microorganisms during first DAIR procedure.

Pathogens	Cases	Control Patients
*Pseudomonas*, *n* (%)	39 (100)	0 (0)
*Enterobacter cloacae*, *n* (%)	1 (2.6)	2 (2.4)
*Escherichia coli*, *n* (%)	6 (15.4)	37 (44.6)
*Escherichia vulneris*, *n* (%)	0 (0)	1 (1.2)
*Klebsiella oxytoca*, *n* (%)	1 (2.6)	9 (10.8)
*Klebsiella pneumoniae*, *n* (%)	2 (5.1)	7 (8.4)
*Proteus mirabilis*, *n* (%)	5 (12.8)	36 (43.4)
*Proteus hauseri*, *n* (%)	1 (2.6)	0 (0)
*Proteus vulgaris*, *n* (%)	1 (2.6)	0 (0)
*Staphylococcus aureus*, *n* (%)	4 (10.6)	29 (34.9)
Other, *n* (%)	22 (56.4)	50 (60.2)

Notes: Other comprises, e.g., Enterobacter species, *Dermabacter* hominis, *Corynebacterium* species, *Streptococcus agalactiae*, *Stenotrophomonas maltophilia*, and *Streptococcus* species. In 10 cases (25.6%) and in 30 control patients (35.7%), *Enterococcus* species were isolated. In 5 cases (12.8%) and 12 control patients (14.3%), Coagulase-negative staphylococci were isolated.

**Table 4:** Oral antibiotic treatment after first DAIR.

Type	Cases	Control Patients
Fluoroquinolones, *n* (%)	39 (100)	84 (100)
Rifampicin, *n* (%)	9 (23.1)	26 (31.0)
Amoxicillin, *n* (%)	6 (15.4)	7 (8.3)
Clindamycin, *n* (%)	5 (12.8)	4 (4.8)
Amoxicillin/clavulanic acid, *n* (%)	2 (5.1)	5 (6.0)
Linezolid, *n* (%)	2 (5.1)	3 (3.6)
Cotrimoxazol, *n* (%)	1 (2.6)	3 (3.6)
Other, *n* (%)	3 (7.7)	13 (16.2)

Notes: Other comprises, e.g., Cefazolin, Minocycline, Ceftazidim, Metronidazole.

## Data Availability

Data are the property of the Department of Orthopaedic Surgery & Trauma, Catharina Hospital, Eindhoven, The Netherlands. Any requests should be directed to Wai-Yan Liu (corresponding author).

## Abbreviations

The following abbreviations are used in this manuscript:

PJI	Periprosthetic Joint Infection
DAIR	Debridement, Antibiotics, and Implant Retention
RIC	Regional Infection Cohort
NINJA	Northern Infection Network Joint Arthroplasty

## Appendix A

**Table A1:** All combinations of isolated microorganisms during first DAIR procedure.

Pathogens	Cases	Control Patients
*Pseudomonas* + *Proteus hauseri* + Other	1	
*Pseudomonas* + *Klebsiella pneumoniae* + Other	1	
*Pseudomonas* + Other	15	
*Pseudomonas* + *Escherichia coli*	5	
*Pseudomonas* + *Proteus mirabilis*	3	
*Pseudomonas* + *Klebsiella pneumoniae* + *Proteus mirabilis*	1	
*Pseudomonas* + *Staphylococcus aureus*	2	
*Pseudomonas* + *Klebsiella oxytoca* + *Proteus mirabilis*	1	
*Pseudomonas* + *Staphylococcus aureus* + Other	2	
*Pseudomonas* + *Proteus vulgaris* + Other	1	
*Pseudomonas* + *Enterobacter cloacae*	1	
*Pseudomonas* + *Escherichia coli* + Other	1	
*Enterobacter cloacae* + *Klebsiella pneumoniae* + *Staphylococcus aureus*		1
*Enterobacter cloacae* + *Proteus mirabilis* + *Staphylococcus aureus*		1
*Escherichia coli* + Other		14
*Escherichia coli* + *Proteus mirabilis* + Other		3
*Escherichia coli* + *Proteus mirabilis* + *Staphylococcus aureus* + Other		1
*Escherichia coli* + *Staphylococcus aureus*		6
*Escherichia coli* + *Staphylococcus aureus* + Other		7
*Escherichia vulneris* + *Staphylococcus aureus* + Other		1
*Klebsiella oxytoca* + Other		3
*Klebsiella oxytoca* + *Proteus mirabilis* + Other		1
*Klebsiella oxytoca* + *Proteus mirabilis* + *Staphylococcus aureus* + Other		1
*Klebsiella oxytoca* + *Staphylococcus aureus* + Other		2
*Klebsiella pneumoniae* + Other		3
*Klebsiella pneumoniae* + *Proteus mirabilis*+ Other		1
*Proteus mirabilis* + Other		12
*Proteus mirabilis* + *Staphylococcus aureus*		5
*Proteus mirabilis* + *Staphylococcus aureus* + Other		4
